# Maternal Docosahexaenoic Acid Increases Adiponectin and Normalizes IUGR-Induced Changes in Rat Adipose Deposition

**DOI:** 10.1155/2013/312153

**Published:** 2013-03-06

**Authors:** Heidi N. Bagley, Yan Wang, Michael S. Campbell, Xing Yu, Robert H. Lane, Lisa A. Joss-Moore

**Affiliations:** ^1^Division of Nutrition, University of Utah, Salt Lake City, UT 84158, USA; ^2^Department of Pediatrics, University of Utah, Salt Lake City, UT 84158, USA; ^3^Division of Neonatology, University of Utah, P.O. Box 581289, Salt Lake City, UT 84158, USA

## Abstract

Intrauterine growth restriction (IUGR) predisposes to obesity and adipose dysfunction. We previously demonstrated IUGR-induced increased visceral adipose deposition and dysregulated expression of peroxisome proliferator activated receptor-**γ**2 (PPAR**γ**2) in male adolescent rats, prior to the onset of obesity. In other studies, activation of PPAR**γ** increases subcutaneous adiponectin expression and normalizes visceral adipose deposition. We hypothesized that maternal supplementation with docosahexaenoic acid (DHA), a PPAR**γ** agonist, would normalize IUGR adipose deposition in association with increased PPAR**γ**, adiponectin, and adiponectin receptor expression in subcutaneous adipose. To test these hypotheses, we used a well-characterized model of uteroplacental-insufficiency-(UPI-) induced IUGR in the rat with maternal DHA supplementation. Our primary findings were that maternal DHA supplementation during rat pregnancy and lactation (1) normalizes IUGR-induced changes in adipose deposition and visceral PPAR**γ** expression in male rats and (2) increases serum adiponectin, as well as adipose expression of adiponectin and adiponectin receptors in former IUGR rats. Our novel findings suggest that maternal DHA supplementation may normalize adipose dysfunction and promote adiponectin-induced improvements in metabolic function in IUGR.

## 1. Introduction

Intrauterine growth restriction (IUGR) predisposes to adult onset disease. The development of obesity following IUGR is well documented and results from adipose dysfunction [1–3]. While IUGR infants are smaller than their appropriately grown counterparts at birth, their rate of weight accretion is accelerated in childhood and they acquire more adipose tissue. An important concept is that IUGR adipose tissue is dysregulated before the onset of obesity [4, 5]. In addition to increased relative amounts of adipose tissue, adipose deposition in IUGR children favors formation of visceral adipose tissue (VAT) over subcutaneous adipose tissue (SAT) [6–8].

We previously demonstrated that IUGR increases the ratio of VAT to SAT in male adolescent rats, prior to the onset of obesity, with no effect in female rats [9]. The changes in adipose deposition in IUGR were accompanied by increased expression of the adipogenic transcription factor peroxisome proliferator activated receptor-*γ*2 (PPAR*γ*2) in VAT, but not SAT, of male rats [9].

An important transcriptional target of PPAR*γ* is adiponectin [10, 11]. Adiponectin improves insulin sensitivity and normalizes adipose deposition [12]. When mice with excessive VAT deposition overexpress adiponectin, VAT is redistributed to SAT in association with improved metabolic parameters [12]. Interestingly, the same outcome occurs when obese mice are given a PPAR*γ* agonist [12]. PPAR*γ* agonists, such as the long chain fatty acid (FA) docosahexaenoic acid (DHA), improve metabolic disease in both human and animal models and increase PPAR*γ* mediated transcription of targets such as adiponectin [11, 13, 14].

The effects of adiponectin are imparted through one of its two receptors Adiponectin receptor 1 (AdipoR1) and adiponectin receptor 2 (AdipoR2) [15]. AdipoR1 and AdipoR2 have been found to be decreased with obesity leading to reduced adiponectin sensitivity [16]. Additionally, AdipoR1 and AdipoR2 are significantly decreased in adipose tissue of insulin resistant ob/ob mice when compared with control mice [16].

The effects of IUGR on adiponectin and adiponectin receptor expression in the rat are unknown. Also unknown are the effects of maternal DHA supplementation on adipose distribution, as well as expression of PPAR*γ*, adiponectin, and adiponectin receptors in the context of IUGR. Because DHA activates PPAR*γ*, we hypothesized that maternal DHA supplementation would normalize IUGR VAT and SAT levels in association with increased PPAR*γ*, adiponectin, AdipoR1 and AdipoR2 expression in SAT. To test these hypotheses, we used a well-characterized model of uteroplacental-insufficiency-(UPI-) induced IUGR in the rat [9, 17, 18] and developed a model of maternal DHA supplementation in the context of the UPI-induced IUGR rat [19].

## 2. Materials and Methods

### 2.1. Animals

All procedures were approved by the University of Utah Animal Care Committee and are in accordance with the American Physiological Society's guiding principles [20]. IUGR was induced by uteroplacental insufficiency (UPI) in Sprague Dawley rats as previously described [9, 19, 21]. Briefly, on day 19 of gestation, pregnant Sprague-Dawley rats were anesthetized with intraperitoneal xylazine (8 mg/kg) and ketamine (40 mg/kg), and both uterine arteries ligated giving rise to IUGR pups. Control dams underwent identical anesthetic procedures. After recovery, rat dams had ad libitum access to food and water. 

Maternal rats were allowed to deliver spontaneously at term; pups were weighed and litters randomly culled to six, to normalize postnatal nutrition. The pups remained with their mothers, feeding via lactation, until day 21 of postnatal life (d21). At d21, control and IUGR rat offspring underwent euthanasia using a sodium pentobarbital overdose (150 mg/kg), blood and serum were collected, and subcutaneous and retroperitoneal (a representative visceral depot) adipose was immediately harvested and flash frozen in liquid nitrogen. 

### 2.2. Maternal DHA Supplementation

DHA was administered via a custom diet [19]. The diet, based on Harlen Teklad 8640 standard rodent diet (TD.8640, Harlan-Teklad, WI), substitutes 1% of the soybean oil in the standard chow with 1% purified DHA (cis-docosahexaenoic acid, no. U-84-A, Nu-Chek Prep, MN). The resulting diet (here called 1% DHA diet) contains the same macronutrient content as standard rodent chow (21.8% protein, 40.8% carbohydrate, and 5.4% fat, with a resulting caloric density of 3 Kcal/g). DHA at 1% was chosen based on our previous studies demonstrating that maternal supplementation with 1% DHA was sufficient to increase pup DHA levels and to be associated with alterations in PPAR*γ* in other tissues [19]. 

The pregnant rats were pair-fed regular diet or 1% DHA diet from E13 and through lactation. The continuation of the maternal diet is important to this study as the fatty acid composition of the maternal milk reflects the maternal dietary fatty acid profile [22, 23]. Each group (Control, IUGR and DHA-IUGR) consisted of 6 male and 6 female pups derived from different litters. 

### 2.3. Magnetic Resonance Imaging

Magnetic resonance imaging (MRI) experiments were conducted using a Bruker Biospec 70/30 instrument (Billerica, MA) and a 72 mm-diameter birdcage radiofrequency (RF) transmit-receive resonator as previously described [9]. ImageJ macros were created to automate the process of image analysis while still allowing manual intervention at key steps that were less amenable to automation [9, 24]. 

### 2.4. Serum Adiponectin Quantification

Serum adiponectin was quantified using an enzyme linked immunosorbent assay (ELISA) (Alpco Diagnostics, NH, (44-ADPRT-E01)) according to the manufacturer's instructions.

### 2.5. Real-Time RT-PCR

Real-time reverse transcriptase polymerase chain reaction (RT-PCR) was used to evaluate mRNA levels in subcutaneous and visceral adipose tissue of adiponectin and adiponectin receptors as previously described [9, 19, 25]. The following Assay-on-demand primer/probe sets were used: PPAR*γ*2-Rn00440940_m1, adiponectin-Rn00595250 m1, AdipoR1-Rn01483784 m1, and AdipoR2-Rn01463173 m1 (Applied Biosystems, CA, USA). GAPDH was used as an internal control (GAPDH primer and probe sequences; Forward: CAAGATGGTGAAGGTCGGTGT; Reverse: CAAGAGAAGGCAGCCCTGGT; Probe: GCGTCCGATACGGCCAAATCCG). 

### 2.6. Protein Analysis

Adipose tissue levels of PPAR*γ*2, AdoR1, and AdoR2 protein were quantified using immunoblotting as previously described [9, 19]. The following primary antibodies were used: PPAR*γ* (H-100, sc-7196, Santa Cruz Biotechnology), AdipoR1 (Santa Cruz Biotechnology, CA, sc-46748), and AdipoR2 (Alpha Diagnostic International, TX, ApidoR21-A).

### 2.7. Statistics

Data are presented as IUGR or DHA-IUGR relative to sex-matched controls ± SEM. Statistical significance was determined using ANOVA using the StatView 5 software package (SAS Institute, Inc.). *P* ≤ 0.05 was considered significant.

## 3. Results

### 3.1. Body Weights

**Figure 1:**
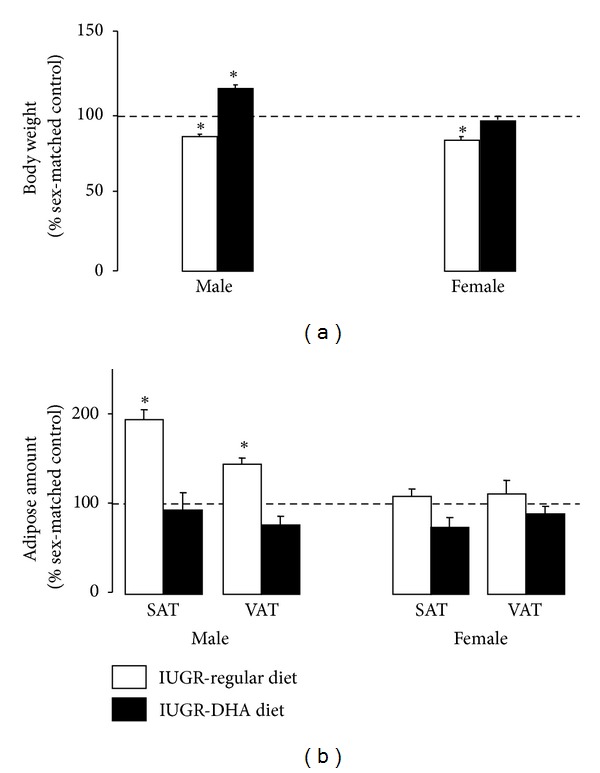
IUGR and DHA-IUGR body weight and adipose deposition. (a) IUGR decreased body weights of male and female adolescent day 21 rats. DHA-IUGR increased male body weight and normalized female body weight compared to controls. (b) Quantification of MRI images. IUGR increased SAT and VAT in male rats. DHA-IUGR normalized SAT and VAT levels in male rats. Sat and VAT deposition was not affected by IUGR or DHA-IUGR in female rats. Results are IUGR (white bars) or DHA-IUGR (black bars) relative to sex-matched controls (represented by the dotted line). Errors are SEM. SAT is subcutaneous adipose tissue, VAT is visceral adipose tissue, *P ≤ 0.05.

Control rat dams supplemented with DHA did not differ in body weight from control rat dams fed a regular diet. Similarly, IUGR rat dams supplemented with DHA did not differ in body weight from IUGR rat dams fed a regular diet. DHA diet also did not affect newborn pup weights for pups from either control dams or IUGR dams (Table 1). Consistent with previously published findings [9], IUGR pups weighed significantly less than control pups through d21. DHA-IUGR significantly increased body weight of male pups and normalized weight of female pups relative to regular diet controls (Figure 1(a)).

### 3.2. Adipose Tissue Distribution

Relative levels of VAT (retroperitoneal) and SAT were quantified from MRI images in male and female control, IUGR and DHA-IUGR rats at d21. Consistent with our previously published study, in male rats, IUGR increased levels of both SAT and VAT relative to regular diet control (*P* ≤ 0.05). In female rats, IUGR did not significantly alter levels of VAT or SAT (Figure 1(b)). DHA-IUGR did not significantly alter levels of VAT or SAT in either male or female rats relative to regular diet controls (Figure 1(b)). 

### 3.3. PPAR*γ*2 mRNA and Protein

**Figure 2:**
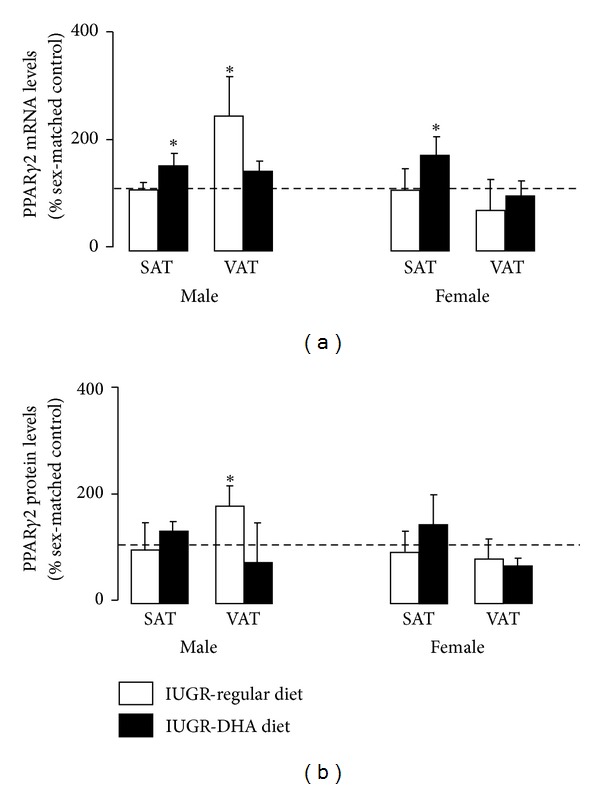
PPAR*γ*2 mRNA and protein levels. IUGR increases VAT PPAR*γ*2 mRNA and protein levels in male rats. DHA-IUGR normalized male VAT PPAR*γ*2 levels and increased SAT PPAR*γ*2 mRNA levels male and female rats. Results are IUGR (white bars) or DHA-IUGR (black bars) relative to sex-matched controls (represented by the dotted line). Errors are SEM. SAT is subcutaneous adipose tissue, and VAT is visceral adipose tissue, *P ≤ 0.05.

Consistent with our previous study, PPAR*γ*2 mRNA was unchanged in SAT and increased in VAT of male rats (*P* < 0.05) relative to male control rats. PPAR*γ*2 mRNA was unchanged in female SAT or VAT relative to female control rats. PPAR*γ*2 protein abundance was similarly increased in the VAT of male rats relative to male control. PPAR*γ*2 protein abundance was unchanged in SAT or VAT of IUGR females. DHA-IUGR significantly increased PPAR*γ*2 mRNA in SAT of male (*P* ≤ 0.05) and female (*P* < 0.05) rats relative to sex-matched controls. DHA-IUGR did not significantly alter PPAR*γ*2 protein abundance in male or female SAT or VAT (Figure 2).

### 3.4. Serum Adiponectin

**Figure 3:**
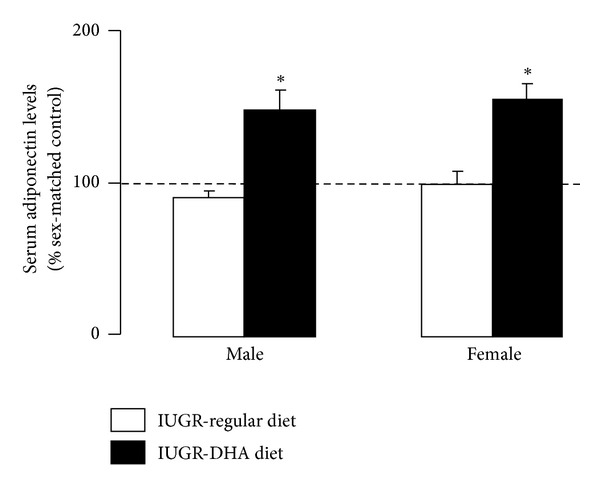
Serum adiponectin levels. DHA-IUGR increased serum adiponectin levels in male and female rats. Results are IUGR (white bars) or DHA-IUGR (black bars) relative to sex-matched controls (represented by the dotted line). Errors are SEM. *P ≤ 0.05.

IUGR did not affect serum adiponectin levels in either male or female rats relative to sex-matched controls. However, DHA-IUGR significantly increased serum adiponectin levels in male and female (*P* < 0.05) rats relative to sex-matched regular diet controls (Figure 3).

### 3.5. Adiponectin mRNA

**Figure 4:**
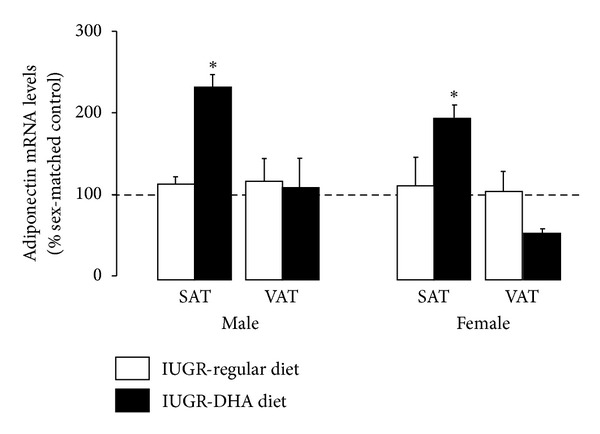
Adiponectin mRNA levels. DHA-IUGR increased SAT adiponectin mRNA levels in male and female rats. Results are IUGR (white bars) or DHA-IUGR (black bars) relative to sex-matched controls (represented by the dotted line). Errors are SEM. SAT is subcutaneous adipose tissue, and VAT is visceral adipose tissue *P ≤ 0.05.

Adiponectin mRNA was unaffected by IUGR in SAT and VAT of male or female rats. DHA-IUGR significantly increased adiponectin mRNA in SAT of male (*P* ≤ 0.05) and female (*P* < 0.05) rats relative to regular diet controls. DHA-IUGR did not affect VAT levels of adiponectin mRNA in male or female rats (Figure 4).

### 3.6. AdipoR1 mRNA and Protein

**Figure 5:**
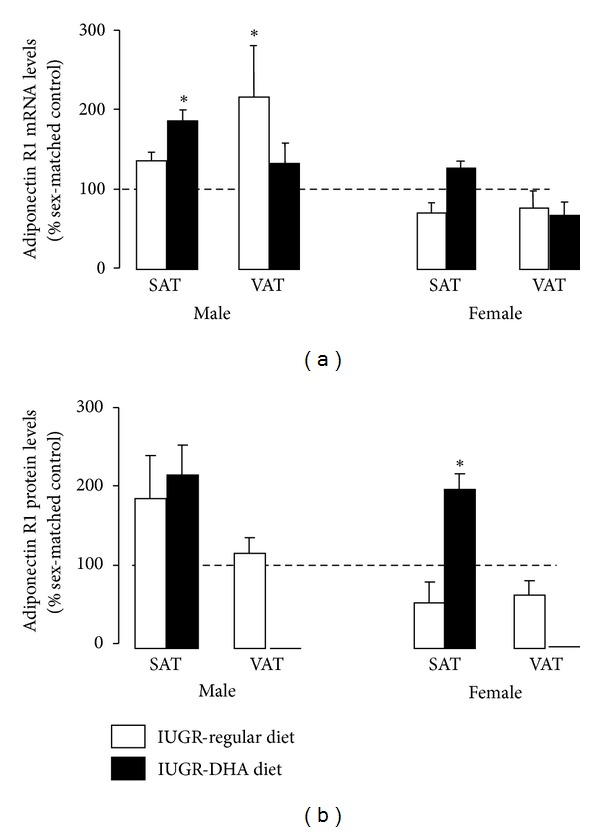
AdipoR1 mRNA and protein levels. IUGR increases VAT AdipoR1 mRNA levels in male rats. DHA-IUGR normalized male VAT AdipoR1 mRNA levels and increased SAT AdipoR1 levels male and female rats. Results are IUGR (white bars) or DHA-IUGR (black bars) relative to sex-matched controls (represented by the dotted line). Errors are SEM. SAT is subcutaneous adipose tissue, and VAT is visceral adipose tissue. *P ≤ 0.05.

In male rats, IUGR significantly increased AdipoR1 mRNA in VAT (*P* ≤ 0.05) relative to male control VAT. IUGR did not alter AdipoR1 mRNA in SAT and VAT of female rats. AdipoR1 protein abundance was also unchanged by IUGR in SAT and VAT of male or female rats relative to sex-matched controls. DHA-IUGR significantly increased AdipoR1 mRNA in male SAT (*P* ≤ 0.05) relative to male control SAT. DHA-IUGR did not affect AdipoR1 mRNA levels of female rats in VAT or SAT. DHA-IUGR significantly increased AdipoR1 protein levels in female SAT (*P* ≤ 0.05), with a trend towards significance in male SAT (*P* = 0.06). Levels of AdipoR1 protein were undetectable in DHA-IUGR rat male and female VAT (Figure 5).

### 3.7. AdipoR2 mRNA and Protein

**Figure 6:**
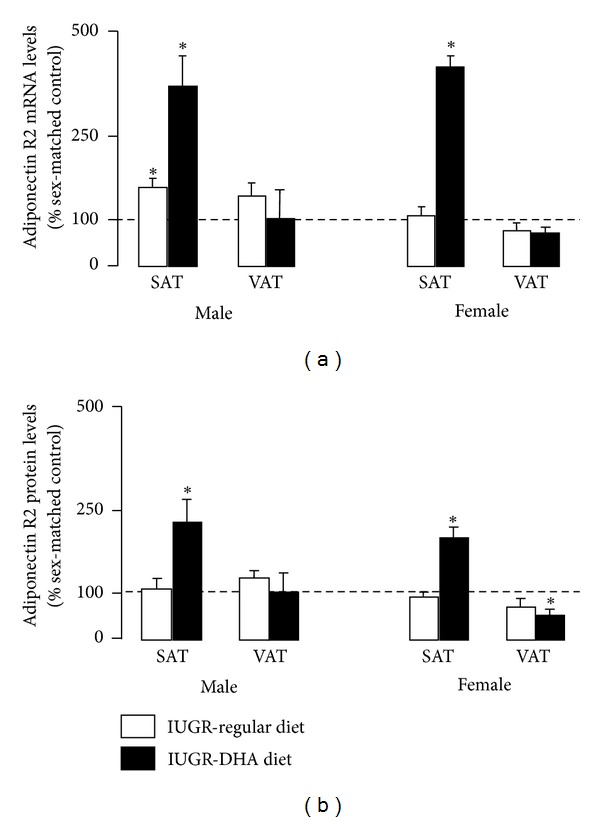
AdipoR2 mRNA and protein levels. IUGR increases SAT AdipoR2 mRNA levels in male rats. DHA-IUGR increased SAT AdipoR2 mRNA and protein levels in male and female rats. Results are IUGR (white bars) or DHA-IUGR (black bars) relative to sex-matched controls (represented by the dotted line). Errors are SEM. SAT is subcutaneous adipose tissue, and VAT is visceral adipose tissue, *P ≤ 0.05.

In male rats, IUGR significantly increased AdipoR2 mRNA in SAT (*P* < 0.05) relative to male control SAT. AdipoR2 mRNA was unaffected by IUGR in SAT or VAT of female rats. AdipoR2 protein was unaffected by IUGR in SAT and VAT of male or female rats. DHA-IUGR increased AdipoR2 mRNA in male (*P* ≤ 0.05) and female (*P* ≤ 0.05) SAT. DHA-IUGR AdipoR2 protein abundance was increased in male (*P* < 0.05) and female (*P* < 0.05) SAT (Figure 6).

## 4. Discussion 

Important findings of our study are twofold. Firstly, maternal DHA supplementation during rat pregnancy and lactation normalizes IUGR-induced changes in adipose deposition and visceral PPAR*γ* expression in male rats. Secondly, maternal DHA supplementation increases serum adiponectin, as well as adipose expression of adiponectin and adiponectin receptors in former IUGR rats. Our novel findings suggest that maternal DHA supplementation may normalize adipose dysfunction and promote adiponectin-induced improvements in metabolic function in IUGR. 

Individuals born IUGR develop adult obesity, with preferential VAT deposition, and insulin resistance [8, 26, 27]. Obesity, preferential VAT deposition, and insulin resistance have been demonstrated in IUGR animal models by our group and others [9, 28–31], often with a sex-specific bias [9, 29, 31]. A common theme amongst IUGR animal models is dysfunctional adipose gene expression that occurs prior to the onset of overt obesity. Our previous observation of increased VAT deposition in adolescent IUGR male rats is consistent with the subsequent development of insulin resistance in this model. This is because VAT and SAT contribute differently to the development of insulin resistance, with VAT being detrimental and SAT potentially conferring protective roles [12, 32, 33]. Normalization of VAT levels in IUGR males following maternal DHA supplementation may be metabolically protective in IUGR. Interestingly, while VAT levels normalized and SAT levels did not increase in IUGR-DHA male rat pups, DHA-IUGR male body weight was increased in our study. This may represent alterations in lean body mass accretion in DHA-IUGR pups.

Supplementation with DHA is physiologically relevant in IUGR. A maternal plasma FA profile low in DHA has been shown to be associated with small for gestational age (SGA) and preterm birth. Additionally, maternal supplementation with DHA during gestation is associated with longer gestation duration [34–36]. In our IUGR rat model, serum DHA is decreased in male neonatal rat pups compared to control, with no significant difference seen in females [19]. The addition of DHA to the maternal diet increases serum DHA levels in male and female DHA-IUGR relative to control [19]. In our model, improved DHA status normalizes VAT PPAR*γ*2 levels in male IUGR rats and increases SAT PPAR*γ*2 levels in both male and female IUGR rats. These findings are consistent with results of PPAR*γ* activation in obese mice. In ob/ob mice, the synthetic PPAR*γ* agonist, Rosiglitazone, results in a redistribution of adipose from VAT to SAT stores, with a concomitant resolution of metabolic disturbances [12]. It is important to note however, that DHA may also help normalize adipose physiology by contributing to other mechanisms, including inhibition of inflammatory pathways [37, 38].

There is evidence that adiponectin may be the link between SAT PPAR*γ* activation and subsequent ablation of metabolic disturbances. Adiponectin expression and release are higher in SAT compared with VAT [39]. When systemically activated, SAT PPAR*γ* prevents adipocyte hypertrophy by increasing the number of small adipocytes which increases adiponectin levels [40]. In ob/ob mice, overexpression of adiponectin produces a similar effect to that of PPAR*γ* activation, with adipose redistribution from VAT to SAT and normalization of metabolic disturbances [12]. Adiponectin deficient mice have increased insulin resistance [41, 42]. Interestingly, obese humans express significantly lower levels of adiponectin even though adiponectin is expressed solely by adipose tissue [43]. Of note, children who are born IUGR also have lower circulating adiponectin at birth [43, 44]. Increased serum adiponectin, especially in individuals who have a predisposition to be insulin resistant, such as those born IUGR, may improve insulin sensitivity. 

In addition to increased serum adiponectin, maternal DHA supplementation increased expression of AdipoR1 and AdipoR2 in SAT in our study. AdipoR1 and AdipoR2 play physiologically important roles in the regulation of insulin sensitivity and glucose metabolism *in vivo* [45]. Obese, insulin resistant mice have decreased expression of adiponectin receptors in adipose tissue (23). The decreased expression of adiponectin receptors is associated with a reduction in the insulin-sensitizing effects of adiponectin. Increasing AdipoR1 and/or AdipoR2 may enhance adiponectin binding capacities in adipose cells and in turn may lead to an increase in the adiponectin effects, even without change in adiponectin levels [46]. Overexpression of AdipoR1 and AdipoR2 in ob/ob mice normalizes insulin signaling [47]. Therefore, DHA-induced increases in both adiponectin as well as its receptors observed in this study may lead to the greatest degree of insulin sensitizing through adiponectin signaling mechanisms.

Our study is not without limitations. First, while we assessed the expression of adiponectin and AdipoR's in adipose tissue, we did not assess adiponectin signaling. An understanding of the downstream consequences of AdipoR activation in IUGR with and without DHA supplementation is important and warrants further investigation. Secondly, while we demonstrated improvement in adipose deposition and increased adiponectin with DHA supplementation in IUGR, we did not assess the effect of DHA supplementation on the development of insulin resistance in this model. Future studies are required to assess the effects of maternal DHA supplementation during gestation and lactation on the development of insulin resistance in IUGR rats. We also did not differentiate between prenatal DHA effects and postnatal DHA effects. Determining the minimum window in which DHA may be effective will be important to enhance the translational relevance of our study.

In conclusion, maternal DHA supplementation increases adiponectin and adiponectin receptor expression in SAT of IUGR rats. We speculate that increased adiponectin production may improve insulin sensitivity in IUGR rats. Our study suggests that early DHA supplementation may provide a means of tempering adipose dysregulation and subsequent metabolic disturbances in IUGR individuals. 

## Figures and Tables

**Table 1:** Maternal DHA supplementation did not alter control or IUGR dam or pup weights relative to regular diet (mean g ± SD).

	Maternal weight		Pup weight	
	Control	IUGR	Control	IUGR
Regular diet	383 ± 34	294 ± 12	6.4 ± 0.6	5.6 ± 0.2
DHA diet	349 ± 18	295 ± 16	6.3 ± 0.3	5.5 ± 0.6
